# Westminster Medical Society, October 21, 1848

**Published:** 1848-12

**Authors:** 


### WESTMINSTER MEDICAL SOCIETY, OCTOBER 21, 1848.

The Society commenced its meetings for the session this evening. The rooms in Saville-row were completely crowded, reminding us of the Society in its most palmy days. About sixty fellows and visitors were present.

The President, on taking the chair, gave an inaugural address on the state of the Society, which was in every way prosperous. In the course of his remarks, he made the following observations with reference to

*Cholera, and the Health of London.*—Before dismissing the subject of cholera, it must be interesting for the fellows to know, notwithstanding the anxiety now prevalent respecting that malignant disease, that hitherto it has not made much progress in the metropolis; and if its present fatality be compared with other epidemic maladies, we have as yet really not much reason for alarm, as proved by the fact, that during the six weeks ending on Saturday, the 16th of October, last year, the number of fatal cases of this disease in London was twenty-six; whilst the total number of deaths caused by the same malady throughout the entire metropolitan population, during the six weeks ending on Saturday last, the 14th instant, amounted to sixty-seven, being, as yet, only a little more than double the mortality by cholera during the same number of weeks in the previous year. Compared with this, it is instructive to mark the different results observed in another epidemic now prevailing in London with great severity, but which, notwithstanding, does not call forth much remark, or cause anxiety to the extent it deserves—I mean scarlatina, also discussed last year in the Society; but which, unfortunately, is now so malignant, that hundreds of victims have been recently sent to an untimely grave, according to the registrar-general's reports. In these tables it is stated, that during the six weeks terminaeing on Saturday, the 16th of October, 1847, already quoted in reference to cholera, 302 individuals died in London from scarlatina; whereas, during the six weeks ending on Saturday last, the 14th inst., as many as 972 persons have sunk under that virulent complaint; or upwards of quadruple the average mortality by the same disease in the previous five autumns. Without undervaluing the importance of the epidemic which at present attracts so much notice, I think such a dangerous malady as scarlatina deserves even as great attention from medical men and the public as cholera—more especially seeing the subjects of its attacks are usually children, or young people just entering upon the morning of life; whereas the victims of cholera are generally drunkards and persons of worn-out constitutions, or those who have set every hygienic rule at defiance. Scarlatina being, however, a disease of frequent occurrence in this country, and although it annually carries off thousands of individuals, hitherto no boards of health have existed; no quarantine laws, and very few sanitary measures, have beenput in force by public bodies for preventing the approach of this malady, the scourge of youth, notwithstanding its highly infectious nature. But this is only another illustration of the prevailing disposition, in the minds of many persons, to view whatever is familiar with indifference, whilst anything new or uncommon is sure to attract attention. It will also be instructive to recall to our recollection the recent invasion of the epidemic influenza, which was so fatally prevalent in the metropolis at the early part of last winter, when 1213 persons died from that complaint during six weeks ending on Saturday, the 8th of January last. At the same time, the total deaths registered from all causes were increased to an extraordinary extent, being so high as 2454 in one week, and 2416 in the subsequent—instead of 1046, the ordinary weekly average of previous seasons. Contrasted with this plague-like mortality, it must be gratifying to hear, that London, comparatively speaking, is not at present unusually unhealthy, notwithstanding the actual presence of cholera, the great malignity of scarlatina, and the prevalence of typhus, by which disease 424 persons have died in the metropolis during the last six weeks, instead of 260, the averaged deaths by typhus of a similar period during the five preceding autumns. Such facts are important; and although the cholera now occasions considerable anxiety, the total deaths from all causes, throughout the metropolitan population, have actually diminished, especially during the last fortnight, notwithstanding the prevalent epidemics. This satisfactory state of the public health in London is proved by the mortality tables, which show, that instead of the weekly average of 1154 deaths, as in the last five seasons, during the week ending on Saturday, the 7th October instant, 1005 persons died from all causes in the metropolis, and only 991 in the week terminating last Saturday, the 14th: thus making an actual diminution of not less than 312 deaths in the two weeks now referred to, being an increase of fifteen and a half per cent. last year over the two similar weeks of the present season. I now mention these important facts to the Society, not to paralyze exertion, but as useful statistical data, to which reference should be made in order to arrive at correct conclusions when an epidemic like the cholera prevails in the community; and to show how far the average mortality is thereby affected.—*London Lancet.*

